# Assessment of a livestock GPS collar based on an open‐source datalogger informs best practices for logging intensity

**DOI:** 10.1002/ece3.4094

**Published:** 2018-05-07

**Authors:** Devan Allen McGranahan, Benjamin Geaumont, Jonathan W. Spiess

**Affiliations:** ^1^ School of Natural Resource Sciences North Dakota State University Fargo North Dakota; ^2^ Hettinger Research Extension Center Hettinger North Dakota

**Keywords:** animal tracking, Arduino, behavioral ecology, DIY ecology, space use patterns

## Abstract

Ecologists have used Global Positioning Systems (GPS) to track animals for 30 years. Issues today include logging frequency and precision in estimating space use and travel distances, as well as battery life and cost. We developed a low‐cost (~US$125), open‐source GPS datalogger based on Arduino. To test the system, we collected positions at 20‐s intervals for several 1‐week durations from cattle and sheep on rangeland in North Dakota. We tested two questions of broad interest to ecologists who use GPS collars to track animal movements: (1) How closely do collared animals cluster in their herd? (2) How well do different logging patterns estimate patch occupancy and total daily distance traveled? Tested logging patterns included regular logging (one position every 5 or 10 min), and burst logging (positions recorded at 20‐s intervals for 5 or 10 min per hour followed by a sleep period). Collared sheep within the same pasture spent 75% of daytime periods within 51 m of each other (mean = 42 m); collared cattle were within 111 m (mean = 76 m). In our comparison of how well different logging patterns estimate space use versus constant logging, the proportion of positions recorded in 1‐ and 16‐ha patches differed by 2%–3% for burst logging and 1% for regular logging. Although all logging patterns underestimated total daily distance traveled, underestimations were corrected by multiplying estimations by regression coefficients estimated by maximum likelihood. Burst logging can extend battery life by a factor of 7. We conclude that a minimum of two collars programmed with burst logging robustly estimate patch use and spatial distribution of grazing livestock herds. Research questions that require accurately estimating travel of individual animals, however, are probably best addressed with regular logging intervals and will thus have greater battery demands than spatial occupancy questions across all GPS datalogger systems.

## 1 INTRODUCTION

Civilian research scientists had access to satellite‐based Global Positioning Systems (GPS) by the early 1990s, and use in animal tracking for ecological research was almost immediate (Rodgers, 2001). Technological advancement and greater user computing power has brought a Big Data approach to animal tracking research in ecosystems around the world (Cooke et al., 2017; Kays, Crofoot, Jetz, & Wikelski, 2015). But while the frontiers of GPS‐based animal tracking are exciting, there remains a core set of research questions that rely on GPS methods. For example, ecologists worldwide use GPS to study the spatial patterns of domestic livestock and managed herbivores to measure animal movement and behavioral responses to heterogeneous environments (Allred et al., 2013; Girard, Bork, Nielsen, & Alexander, 2013; Raynor et al., 2017; Zhao & Jurdak, 2016). Data from animal‐borne GPS receivers can also enhance agroecosystem sustainability by giving managers information useful to increase productivity and identify areas of use sensitive to environmental degradation (Haan, Russell, Davis, & Morrical, 2010; Turner, Udal, Larson, & Shearer, 2000). Not only is domestic livestock management a global industry and foundation of rural livelihoods (Randolph et al., 2007), it is also an important component of natural areas management as a prescribed approach to ecological disturbance (Pietzsch, Ochsner, Mantilla‐Contreras, & Hampicke, 2013). Thus, whether for production or conservation grazing, GPS technology is an important resource for monitoring patterns of livestock space use patterns (Allred, Fuhlendorf, & Hamilton, 2011; Putfarken, Dengler, Lehmann, & Härdtle, 2008).

Despite the benefits of fine‐scale insight into herbivore space use, logistical constraints such as cost and functionality limit access to GPS tracking technology for many potential users. Commercially available systems can be so expensive that a recent criterion for a “low‐cost” solution was under US$1,000 (Clark et al., 2006). While several times less costly than many commercial systems, US$1,000 is still prohibitively expensive for users in the developing world who stand to benefit from GPS tracking data, such as herder communities in Ethiopia and Morocco (Akasbi, Oldeland, Dengler, & Finckh, 2012; Liao, 2017).

Even those with Western research budgets face questions about how to best use GPS technology in ecological studies. GPS‐based tracking systems pose trade‐offs between data quality and quantity, hardware capability, and cost. Initially, data quality issues related to the accuracy of the receiver unit, and quantity depended on data storage capacity within the span of battery life. Ensuring maximum accuracy, storage capacity, and battery life was expensive, and lower‐cost systems required compromise on one or more facets. As receiver accuracy has improved and data capacity has increased dramatically while the size and cost of storage units have decreased, data quality is now more a question of temporal resolution to address more complicated questions about animal behavior (Johnson & Ganskopp, 2008). For example, estimating the size of wolf home ranges with GPS data is sensitive to logging frequency (Mills, Patterson, & Murray, 2006), but the difficulty in fitting GPS collars onto wolves in the first place incentivizes long logging periods.

Another issue related to sampling frequency is how different logging intervals affect estimations of total distance traveled by collared animals. Less linear, more tortuous paths are underestimated by infrequent logging intervals, which cut corners and return low accumulated distance (Johnson & Ganskopp, 2008; Marcus Rowcliffe, Carbone, Kays, Kranstauber, & Jansen, 2012). Thus, one might conclude that the major limitation in GPS receiver technology today is battery life: Batteries are large, heavy, and expensive, but for many systems logging positions more frequently draws batteries down.

We sought an ultra‐low‐cost GPS animal tracking solution, and through the process of development inform outstanding questions about herd‐level replication and logging intervals. We use hardware and software based on Arduino (https://www.arduino.cc), an open‐source electronics platform. Designed for physical computing, Arduino consists of low‐cost, pre‐assembled boards programmed with a freely available, cross‐platform, open‐source Integrated Development Environment (IDE) that uses C/C++ programming languages. Most of the functions users need are provided with the IDE or are freely available online from third‐party developers.

While open‐source microcontroller systems–including Arduino–have been adopted in both the field and laboratory (Barnard, Findley, & Csavina, 2014; Greenspan et al., 2016; Shipley, Kapoor, Dreelin, & Winkler, 2017), we found no instances of Arduino‐based GPS tracking systems in peer‐reviewed literature. Here, we report on the assembly, programming, geolocation performance, and field deployment of Arduino‐based GPS dataloggers on two different types of grazing animals, cattle and sheep. We use data from the trials to address two questions of general importance across GPS logger systems, not just those based on Arduino: (1) How closely do collared animals in a herd track together? (2) How do different logging patterns compare to constant, low‐interval logging in terms of measuring both spatial distribution and distance traveled? Respectively, addressing these questions inform issues of within‐herd logger replication (and overall project cost), and optimizing sampling intensity with battery life.

## 2 MATERIALS AND METHODS

### 2.1 The GPS logger system

#### 2.1.1 Basic hardware

The foundation of our system is an open‐source microcontroller based on the Arduino electronics platform. Specifically, our hardware comes from the Adafruit Industries Feather series (https://www.adafruit.com/feather). Adafruit's Feather hardware is compatible with the Arduino Integrated Development Environment (IDE) for programming small electronics projects powered by lithium ion polymer (Li‐Po) batteries for mobility. With pre‐assembled boards fitted with various sensors, receivers, and other peripherals (referred to as FeatherWings) designed as part of the Feather series, even a novice can develop customized electronic solutions with minimal experience in either hardware or software. The components come as simple kits, and users solder header pins to stack wings onto the microcontroller board and seamlessly connect to its circuitry, often with little or no additional wiring. Adafruit provides freely available, open‐source software libraries that integrate easily with the IDE and Arduino programs.

**Figure 1:**
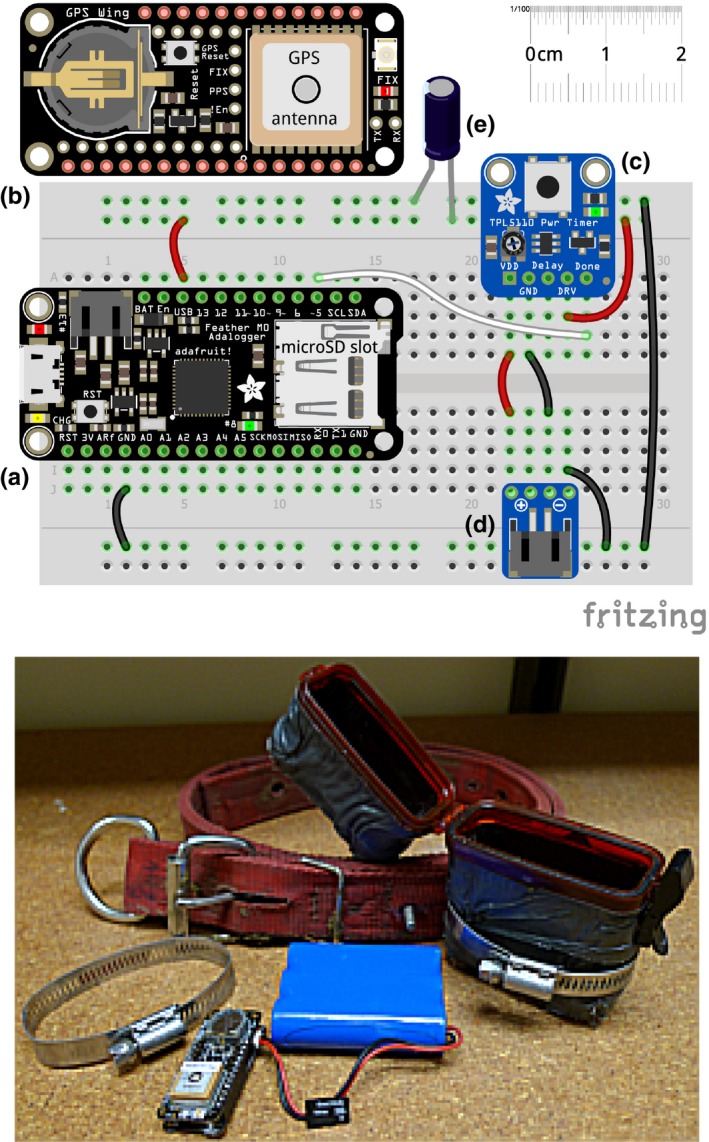
Top: Illustration of our Global Positioning Systems (GPS) datalogger electronics. Basic components include the Adafruit Feather M0 Adalogger (a) and Adafruit GPS Wing (b). Also shown are the TPL5110 low power timer (c), external JST battery plug (d), and a 47uF capacitor (e) to stabilize the power supply. Bottom: The assembled GPS Wing and Adalogger, with battery, case, clamps, and collar

Our system is comprised of three basic components: microcontroller, datalogger, and GPS receiver (Table 1). Adafruit has combined the microcontroller and datalogger into a single board with the Feather M0 Adalogger, which includes a ATSAMD21G18 ARM Cortex M0 microchip and microSD card slot (Figure 1). Using header pins, the FeatherWing GPS receiver simply slides onto the top of the Adalogger. The system is powered by 3.7 v Li‐Po batteries; we used the highest capacity available from Adafruit, 6,600 mAh, for maximum run‐time (approx. 1 week under default configurations of the Adalogger + GPS FeatherWing). These batteries are easily rechargeable via USB chargers.

**Table 1:** Components of livestock GPS collar system designed around the Arduino‐based Feather series by Adafruit Industries. See Figure 1 for connections, layout, and assembly

Component type	Component	Source	Price (USD)
Core hardware	Feather M0 Adalogger	Adafruit Industries	$19.95
	Ultimate GPS FeatherWing	Adafruit Industries	$39.95
Additional components	Lithium ion polymer battery	Adafruit Industries	$8–30
	micro SD card	amazon.com	$5–10
	Lifeline 4430 waterproof ABS case	amazon.com	$3.99
	Nylon livestock collar	Nasco	$9–15
	Rubber splicing tape	Hardware store	$10/roll
	Hose clamps, nuts and bolts, duct tape	Hardware store	$10
	Micro Li‐Po USB battery charger	Adafruit Industries	5.95
	TPL5110 Low Power Timer + JST plug	Adafruit Industries	$6.45
	ADXL335 3‐D accelerometer	Adafruit Industries	$14.95

GPS, Global Positioning Systems.

#### 2.1.2 Attachment to livestock

The GPS dataloggers were deployed on livestock by sealing them in waterproof cases attached to heavy‐duty nylon collars sized for the species and age class of the animals. We found a small plastic case designed for first aid kits—available in outdoor equipment stores and online (Table 1)— that was perfectly suited for the 6,600 mAh battery and stacked Feather logger/receiver. We cut small pieces of styrofoam to fit around the Feathers to reduce motion in the headspace of the case and prevent the microSD card from being ejected. We also included small silica gel packets in the cases to absorb moisture and reduce corrosion on the Feathers. Prior to attachment, we reinforced the cases with rubberized electrical tape and heavy‐duty outdoor duct tape.

**Figure 2:**
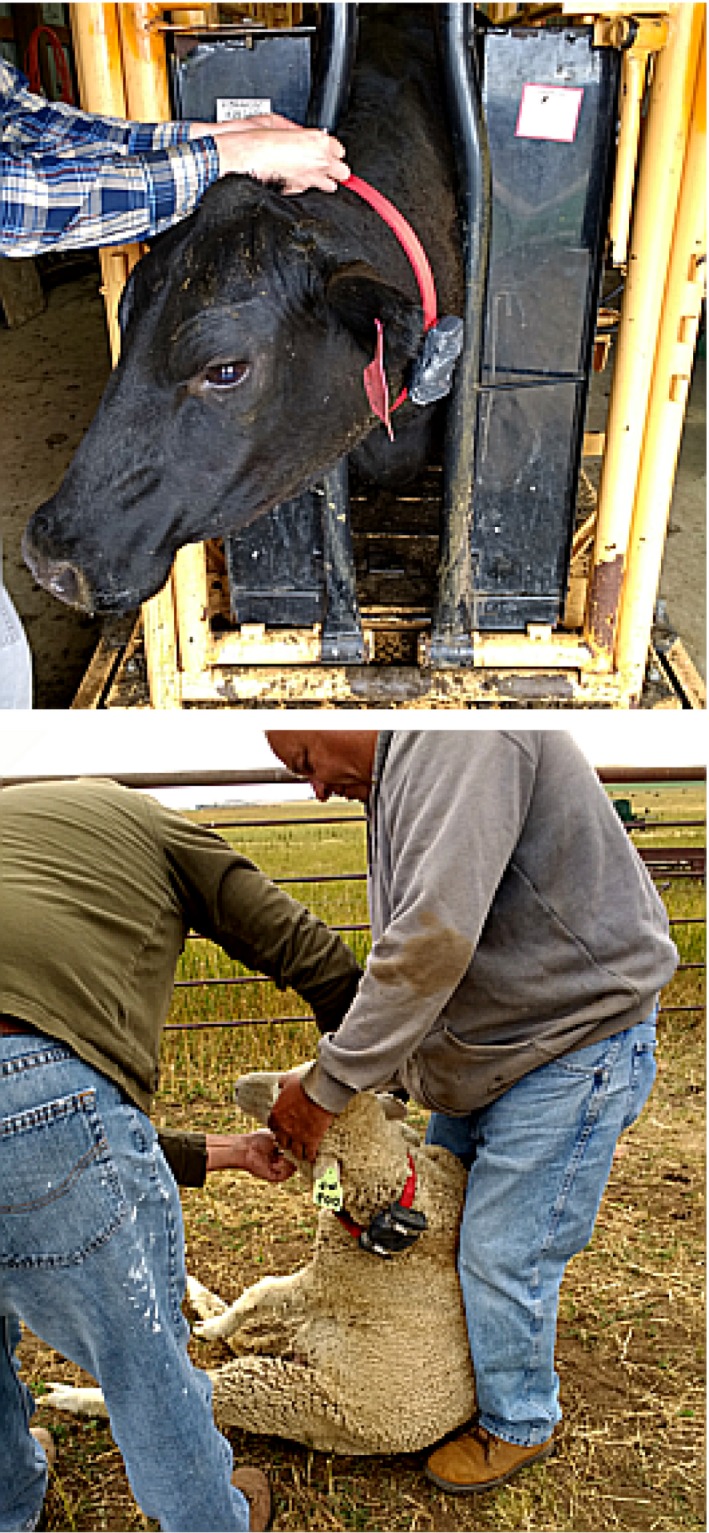
Top: Attaching a collar with duct tape‐wrapped Global Positioning Systems datalogger on a cow ahead of initial release onto experimental pastures in June. Bottom: Retrieving a collar from a sheep in the field

We attached the sealed datalogger cases to the livestock collars with hose clamps. Cattle were restrained in an adjustable headgate while collars were attached, while attachments to sheep were made both in headgates and while held by herders in the field (Figure 2). Collars were fastened at the top of the neck just behind the head, tightly but allowed to rotate. We observed no evidence that the collars or the units—which weighed less than 300 g— impaired animal mobility or behavior. Collars were retrieved by rounding animals up in the field and simply undoing collar fasteners.

### 2.2 Software

#### 2.2.1 Programming the datalogger

We used software examples from Adafruit to program the GPS dataloggers in the Arduino IDE. The basic sketch includes two main subroutines: read and parse location information from the GPS receiver, and write data to the microSD card (Appendix S1). In the GPS subroutine, functions from the Adafruit GPS library, called with Adafruit_GPS.h, get a fix from satellites and parse the data. Data are read once per program cycle, and the user controls logging frequency by defining a delay between program cycles, which we set to record a location every 20 s. Data are handled as strings of ASCII characters formatted as an National Marine Electronics Association (NMEA) sentence parsed by the function GPS.parse.

Within the same program cycle, the next subroutine writes data to the microSD card via the dataFile.print command from the library called by SD.h. The data row consists of specific entries from the parsed NMEA sentence, in any specified order. By printing a comma between each data value and writing a single line for each observation, the program constructs a file comprised of comma‐separated values readable as a.csv file.

#### 2.2.2 Postprocessing data

While the data file created on the microSD card is human‐readable via any basic text editor, spreadsheet application, or data analysis package capable of reading comma‐separated values, one quirk in the raw NMEA data returned by Arduino's GPS.parse function requires postprocessing. The $GPGLL phrase—Geographic Position, Latitude/Longitude, and time— is in the format lll.ll,a, for degrees, decimal minutes, and hemisphere. Take, for illustration, the example from http://aprs.gids.nl/nmea/#gll: Latitude 49 degrees 16.45 min North is stored as 4916.46,N, which is not readily interpretable to data analysis, graphing, and most GIS software. However, this obscure but standard format is easily converted into degrees/minutes/seconds, decimal degrees, or the Universal Transverse Mercator (UTM) coordinate system, for which we provide script written for the freely available R statistical environment (R Core Team 2017) in Appendix S2. We assigned Coordinate Reference Systems in R with functions from the sp package (Pebesma & Bivand, 2005).

Other steps in postprocessing the GPS data included converting the timestamp to a time‐date POSIXct object in R with the strptime function and correcting the default UTC time to local time with the hours function in the lubridate package (Grolemund & Wickham, 2011), which allows one to focus on livestock activity at specific periods within days and seasons. While GIS‐related steps in the analysis are discussed below, we began by cropping GPS locations to the experimental pastures to remove positions beyond the pasture boundaries due to transport of turned‐on but unattached collars, a couple instances of sheep getting through their fence, and very few inadvertent outlier positions likely due to poor satellite fixes.

#### 2.2.3 Precision and accuracy testing

As we could find no information on the geolocation performance of the Adafruit GPS Wing, we modified the protocol of Clark et al. (2006) to measure the average horizontal error or circular error probability (CEP) of our datalogger units: Essentially, we determined how closely positions logged by our dataloggers matched up with a known position on the Earth's surface, specifically, U.S. National Geodetic Survey geodetic survey point RP1098 in Fargo, North Dakota. Dataloggers were arranged on the top of a 1.2‐m fiberglass stepladder directly above the survey point and logged positions at 20‐s intervals for 90 min. From these data, we calculated both precision and accuracy for each unit, defined, respectively, as how tightly positions from a single unit clustered, and how far logged positions were from the known georeference point. For each measure, we calculated CEP as the 95% quantile for each unit (Clark et al., 2006). Distances between individual positions and either the centroid of a unit's positions (precision calculation) or the known georeference (accuracy calculation) were computed with the distGeo function in the geosphere package (Hijmans, 2016) for R.

### 2.3 Field trials

#### 2.3.1 GPS datalogger deployments

We made 4‐week‐long deployments in the summer of 2017 on experimental rangeland at the Hettinger Research Extension Center in Hettinger, North Dakota, which included four, 65 ha pastures, two stocked with cattle and two with sheep. An initial trial in June consisted of three dataloggers deployed to one cattle pasture and another three dataloggers deployed to one sheep pasture. Collars were attached to randomly selected animals, while they were being worked through livestock handling facilities prior to initial release to experimental pastures for the grazing season. Sampling effort was doubled for July, August, and September trials with three units deployed in each of two additional pastures, one cattle and one sheep.

#### 2.3.2 Data management

We developed a workflow for loading and processing Feather GPS data in R (Appendix S2). We begin with a function that loads and combines individual .TXT files written to the microSD card by each Feather M0 Adalogger into a single R data.frame. Then, we remove duplicate entries from the combined data.frame, which occur when data from a previous deployment remain on the microSD card. Subsequent steps convert default NMEA format for latitude and longitude into the UTM coordinate reference system (UTM Zone 13, datum NAD83).

After cropping the positions to pasture boundaries, we combined the positions with a spatial data.frame containing information on location names and management status using the sp package in R. We then discarded locations incorrectly assigned to the wrong pasture by the data.frame merger, which occurred where experimental pastures shared a fence boundary and locations were incorrectly recorded on the wrong side of the fence as animals tracked along it, or in a couple instances, when sheep actually crossed fences into neighboring pastures. For our analyses, we excluded any individual logger deployment that did not log at least 1,000 locations. Full script for analysis is available in Appendix S3.

#### 2.3.3 Data analysis

To inform the minimum number of dataloggers required to estimate spatial distribution of livestock herds, we calculated how closely collared animals within a pasture ranged with respect to each other. We wrote a script to compute distance matrices during the four datalogger deployments using the vegdist function in the vegan package (Oksanen et al., 2017) for the R statistical environment. By calculating distance matrices with the Euclidean distance measure based on UTM coordinates, this function returns the shortest distance between logged positions in meters. Our script used only the first logged position per minute for each datalogger, to minimize variability caused by differences in which points in the minute a given datalogger spaces its 20‐s logging intervals. We excluded nighttime positions from the analysis (22:00–04:00). Our script returned the mean distance among collared animals per minute per pasture, based on a maximum of three dataloggers/pasture. When only two dataloggers had a position in a given minute due to the third registering an outlier or being nonfunctional, the script returned the distance between the two valid points. If a minute contained one or no valid points for a given pasture, the script did not return any distance value for that pasture in that minute.

We compared different patterns and frequencies of logging GPS positions on two measures of animal behavior frequently studied with GPS data: spatial distribution and distance traveled. Our two alternative sampling patterns included regular— in which data were subsampled regularly at 5‐ and 10‐min intervals—and burst– in which all positions from 5‐ and 10‐min durations were sampled from each hour; the combination of 5‐ and 10‐min intervals between regular and burst patterns created four potential ways a datalogger could be programmed to reduce the size of data files and/or extend battery life. Data were subsampled with the cut function to re‐format the timestamp of each position to fall within either a 5‐ or 10‐min window. We then extracted either the first position per window for regular logging, or all positions within the first window of each hour for burst logging.

To compare patterns of spatial distribution, we first calculated the proportion of all daytime locations logged at 20‐s intervals (constant logging) within equal‐sized patches in each pasture, subset the constant logging data as if collected under each of the four patterns, and determined the difference between the proportional distribution of these data within each patch to the distribution of the constantly‐logged data. We used two patch sizes: ~16 ha, created by dividing our ~64 ha pastures into four equal patches; and 1 ha, created by subdividing 16 ha patches into 100 × 100 m grids. Because these divisions created many more 1‐ha patches than 16‐ha patches, and 1‐ha patches are potentially more susceptible to error, we employed a conservative approach and report the maximum difference among all patches per datalogger rather than the mean. We calculated the absolute value of differences to ensure positive and negative differences between the distribution of positions under constant and subsampled logging did not cancel out the magnitude of difference between the patterns.

To compare travel distance, we summed the distance between logged positions for each collared animal per day under each logging pattern and compared them as a percentage of the total distance recorded by constant logging at 20‐s intervals. Distance between logged GPS positions was calculated with the distGeo function in geosphere. To explore the utility of applying correction factors to underestimated travel distance by less‐frequent sampling (e.g., Akasbi et al., 2012), we used the maximum likelihood function mle2 in the bbmle package (Bolker & R Core Team, 2017) to estimate regression coefficients for each combination of logging pattern and interval from linear models. June data were excluded from this first step, so as to retain a novel dataset to which we multiplied estimated distances for each logging pattern by that pattern's estimated correction factor and re‐calculated regression coefficients from the corrected linear model fit to June data. We calculated 95% confidence intervals for the slope coefficients to determine difference from 1; a slope of 1 between distance determined by constant logging and estimated by different logging patterns indicates accurate estimation.

### 2.4 Extending battery life

The constant, high clock speed of the Feather M0's processor drains battery life. Adafruit's hardware solution is the TPL5110 low power timer, which bypasses the direct power supply between battery and board and only supplies power at set durations. Integration with the Feather M0 Adalogger is simple but does require minor external wiring (Figure 1).

Because many Arduino projects take less than a second or two to initialize, external power regulation is an elegant solution, but GPS logging poses a potential pitfall in that satellite fixes are lost when the unit loses power and longer power‐off periods can slow fix re‐acquisition when power is restored. As such, meaningful data require an adequate logging period balanced by a reasonable power‐off period, so GPS receiver initialization is a relatively infrequent event with marginal contributions to power consumption, which motivates our “burst” sampling.

## 3 RESULTS

### 3.1 Unit performance

Mean 95% circular error probability (95% CEP) for all units with respect to the known georeference point—our measure of GPS unit accuracy— was 4.0 m (±0.3 *SE*). Unit precision was 1.8 m (±0.2 *SE*).

Our GPS dataloggers performed well in field trials with limited equipment malfunction. Only two datalogger units were excluded entirely from the dataset due to recording less than 1,000 locations: one due to unrelated sheep mortality, and one when it appeared a battery was incompletely charged. We determined that excessive movement within the case could cause the spring mechanism of the microSD card holder to eject the cards. We cut scrap styrofoam to fit around the Feather units and take up headspace in the case, after which all units logged for at least 109 hr (16,000 valid positions) and up to 190 hr (nearly 32,000 valid positions) per week‐long deployment, with an average of 171 logging hours.

**Figure 3:**
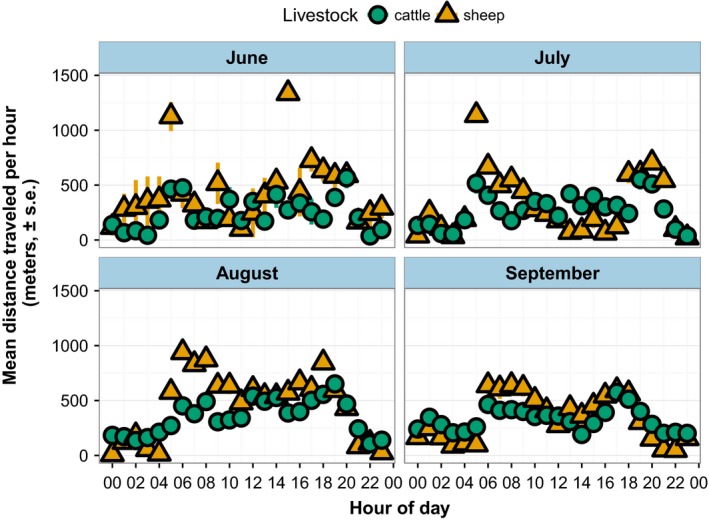
Mean distance traveled per animal, per hour, across all days within each month. Low activity levels between 22:00 and 04:00 justify removing this nighttime period from subsequent analysis. Data from two cattle and two sheep pastures in Hettinger, North Dakota, with 2–3 collared animals per pasture (actual number within each mean varies with individual collar performance and battery life)

Each step in the data processing workflow intended to clean up the GPS data removed rows from the data.frame, but the proportion of positions removed for low‐quality fixes and being beyond pasture boundaries was extremely low. After cleanup, we used 718,510 logged positions in our analysis from a total of 4,962 functioning datalogger hours over 33 successful individual datalogger deployments of 39 deployments attempted. Focusing analysis on positions recorded between 04:00 and 22:00 is justified by low activity levels as evidenced by lower distances traveled than any other period in the day among both cattle and sheep throughout the season (Figure 3).

### 3.2 Distance between units

**Figure 4:**
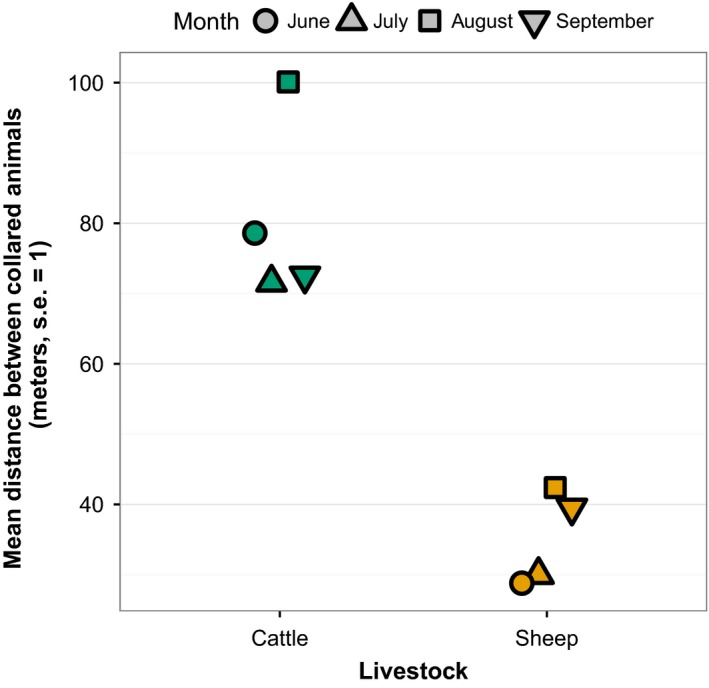
Mean distance between two and three collared animals per pasture between 04:00 and 22:00 during 4‐month‐week‐long Global Positioning Systems datalogger deployments in two cattle and two sheep pastures in Hettinger, North Dakota

Collared animals within the same pasture generally remained close to each other throughout the grazing season (Figure 4). Despite having many more animals per herd, sheep maintained closer distances than cattle: Collared sheep were within 25 m of each other in 50% of all daytime positions versus 64 m for cattle. Sheep spent 75% of the daytime period within 51 m, whereas cattle spent 75% of the daytime period within 111 m. The mean distance among collared animals was greatest among cattle in August (100 m), and otherwise, the mean distance between collared cattle was 72–79 m (Figure 4). Sheep averaged no more than 42 m apart (Figure 4).

### 3.3 Logging pattern comparison

**Figure 5:**
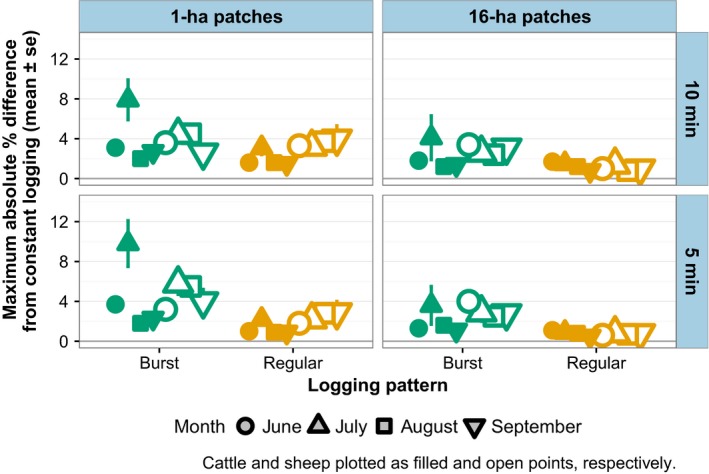
Maximum percentage difference among each datalogger deployment between proportion of locations in each 1‐ and 16‐ha patches under four logging patterns (hourly bursts of 5‐ or 10‐min duration, regular 5‐ or 10‐min intervals) compared to the proportion of locations in each patch under continuous logging. Differences expressed as absolute values. Data consist of positions logged between 04:00 and 22:00 during 4–7‐day trials on two cattle and two sheep pastures in Hettinger, North Dakota

We found very little difference in the proportion of positions recorded in patches within pastures by four different logging patterns compared to constant logging (Figure 5). There was very little difference in the proportion of locations recorded in 1‐ha versus 16‐ha patches. The maximum degree of difference occurred among cattle sampled under the burst logging pattern in July at both 5 and 10‐min durations, which differed from constant logging by an average of 3% and a mean maximum among dataloggers of 8% (Figure 5). Otherwise, the maximum difference in the proportion of positions recorded in a given 1‐ or 16‐ha patch under burst or regular logging did not exceed 6%, with a mean difference of 2%.

**Figure 6:**
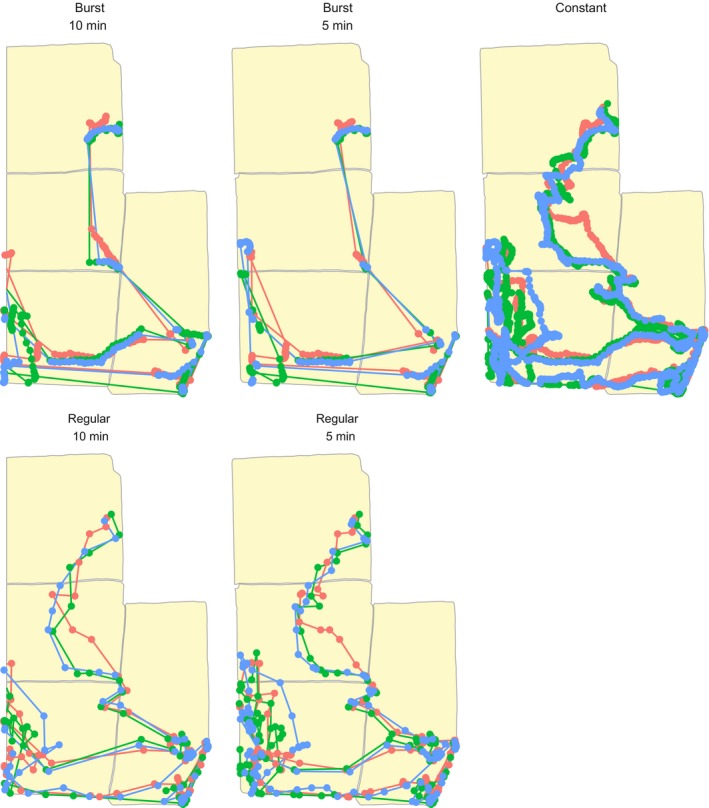
An example of four logging patterns (hourly bursts of 5‐ or 10‐min duration, regular 5‐ or 10‐min intervals) compared to constant logging at 20‐s intervals for three sheep fitted with DIY GPS dataloggers at the Hettinger Research Extension Center, Hettinger, North Dakota. Data are from between 04:00 and 22:00 on 7 July 2017. Pasture divisions represent the patches used to compare space use patterns by the four logging settings (Figure 5). These maps also illustrate how different logging patterns might vary in their estimation of total distance traveled, especially over nonlinear/tortuous routes

Regular logging was the least variable, with no maximum difference from constant logging above 5% (Figure 5) and mean differences always under 1% difference across months and livestock types. Figure 6 provides an example of how the different logging patterns compare to constant logging for one sheep pasture on a randomly selected day in July.

### 3.4 Distance traveled comparison

**Figure 7:**
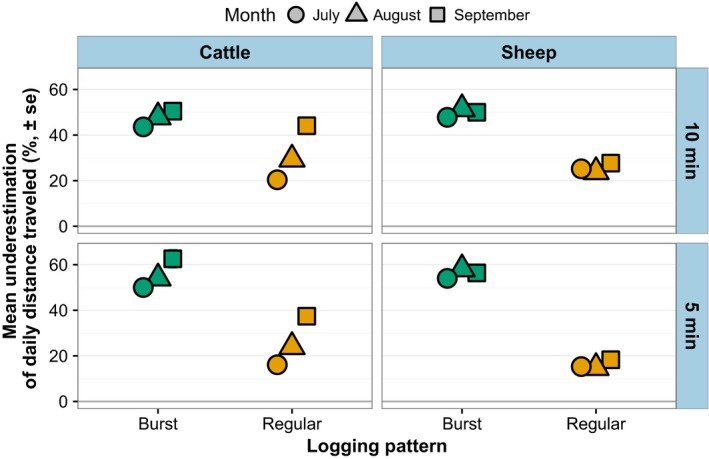
Differences in total daily distance traveled for collared animals under four logging patterns (hourly bursts of 5‐ or 10‐min duration, regular 5‐ or 10‐min intervals) compared to continuous logging. Data consist of positions logged between 04:00 and 22:00 during 3–7‐day trials on two cattle and two sheep pastures in Hettinger, North Dakota

Logging fewer GPS positions resulted in underestimated total daily distance traveled for collared animals, when the four logging patterns were compared against constant (20‐s interval) logging (Figure 7). Not surprisingly, regular logging performed better than burst logging in terms of less severe underestimation of total daily distance. Shorter, 5‐min intervals performed better than 10‐min intervals, especially for sheep. On average, cattle traveled 4,399 m (±201 *SE*) per day, while sheep traveled 5,406 m (±221 *SE*) per day.

**Figure 8:**
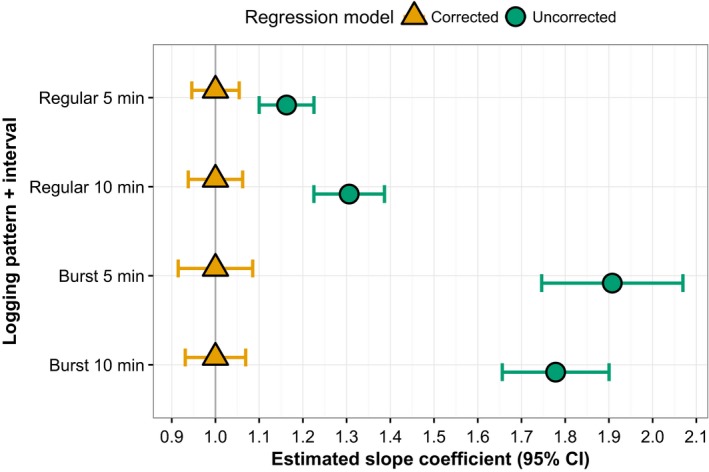
Slope parameters from linear regression models comparing estimated daily distance traveled for collared animals under four logging patterns (hourly bursts of 5‐ or 10‐min duration, regular 5‐ or 10‐min intervals) to continuous logging. Corrected models multiply the estimated distance in June data by the slope parameter from the uncorrected model, which was determined by maximum likelihood estimation on data from July, August, and September; the closer the corrected parameter is to 1, the better the correction factor performs in increasing the accuracy of distance estimates. Data consist of positions logged between 04:00 and 22:00 during 3–7‐day trials on two cattle and two sheep pastures in Hettinger, North Dakota

Using linear regression coefficients from maximum likelihood estimation as correction factors was effective in compensating for underestimations in total daily distance traveled (Figure 8). As indicated by the degree of underestimation (Figure 7), linear models fitting total daily distances from constant logging against those estimated by different logging patterns had regression coefficients substantially above 1.0, with burst logging approaching 2.0 (Figure 8). But in each case, multiplying novel data by these estimated regression coefficients prior to comparison with constant logging produced 95% confidence intervals centered around 1.0, meaning they accurately predicted actual total daily distance traveled.

### 3.5 Battery life extension

Fitting the GPS dataloggers with the TPL5110 low power timer (Figure 1) and programming the unit for the 5‐min burst logging pattern substantially increased battery life in laboratory tests. Use of the low power timer increased battery life by a factor of 7.5, which when applied to our average battery life of 171 logging hours suggests battery life could be extended to as long as 1,306 hr, or 54 days. While actual performance in the field is likely affected by battery age, operating temperature, and time spent re‐acquiring satellite fixes, these results suggest incorporating the power timer might greatly increase the utility of the system for situations where animals can be handled at monthly but not weekly intervals. Alternatively, users satisfied with shorter deployments can use batteries that weigh, and cost, less.

## 4 DISCUSSION

The purpose of this study was twofold: First, we sought to create a low‐cost GPS datalogger with high data storage capacity, high‐frequency logging, and sufficient durability for attachment to rangeland livestock. Second, we used these high‐frequency data to test general questions related to datalogger replication within herds and trade‐offs between battery life and accurately representing animal behavior. The dataloggers functioned well in the field trial, and integrating the TPL5110 low power timer can potentially extend battery life by a factor of seven, which means up to 50 days of burst logging with a 6,600 mAh battery. Our 20‐s logging interval certainly qualifies as high‐frequency for the purposes of comparing error created by less‐frequent intervals: While some studies logged at 10‐s intervals and calculated error from there (Liu, Green, Rodríguez, Ramirez, & Shike, 2015; Swain, Wark, & Bishop‐Hurley, 2008; Zhao & Jurdak, 2016), other studies used baseline data from intervals as long as 4–5 min (Johnson & Ganskopp, 2008; Mills et al., 2006) and 15 min (Akasbi et al., 2012).

We found that collared animals within the same pasture spent most of their time close to one another, suggesting that few GPS datalogger units per herd are necessary to monitor spatial distribution. Collared cattle were, on average, within 76 m of each other, and sheep within 42 m of each other, on pastures approximately 800 × 800 m square. Clearly, sheep herd very tightly; collared sheep were closer together despite their herds being an order of magnitude larger (stocking rates were approximately 25 cattle vs. 175 sheep per pasture). Distances among animals were generally consistent with other research: Schwager, Anderson, Butler, and Rus (2007) showed high variability around mean distances to cattle herd centroids of 50–60 m in 466 ha of arid rangeland, and Guo et al. (2009) showed high variability around mean cow‐cow distances of 20–25 m in small (7 ha) paddocks.

Based on these data, we suggest that one datalogger is probably sufficient to record herd locations at a landscape level, but recommend at least 2–3 to ensure data redundancy in the face of equipment malfunction or animal mortality. Conversely, Liu et al. (2015) suggest up to 75% of animals in a group should be fitted with collars to estimate spatial occupancy, which for most researchers would be cost‐prohibitive: Such a sampling intensity for our sheep herds would require 130 dataloggers *per pasture* for the animals we found to cluster the tightest. At the very least, sufficient sampling intensity likely varies with species, environmental heterogeneity, and research question (Augustine & Derner, 2013). For work in rangelands and wildlands, investigators are likely to gain more information by distributing equipment across replicate herds in heterogeneous environments despite possible loss of accuracy.

Each of our four logging patterns provided low rates of error in determining occupancy of both small (1 ha) and large (16 ha) patches in our experimental landscapes. The accuracy of determining the spatial distribution of animals with GPS data declines as the interval between logged positions increases; hourly intervals produce prediction error rates of 90% for even slow‐moving cattle (Swain et al., 2008). Our maximum error rate was 8%, and averages were around 2%–3%. According to Swain et al. (2008), an under‐appreciated factor in GPS data quality is how quickly the GPS receiver obtains a “fix” on satellites, and they describe a solution very similar to our burst logging pattern that greatly reduces error from slow fixes by remaining on and connected to satellites for the duration of the logging period.

Total daily distance traveled by collared animals in our study was consistent with other data published from rangeland systems. Lomillos Pérez, Alonso la Varga, García, and Gaudioso Lacasa (2017) reported cattle in rangeland pastures traveled an average of 3.3 km per day during warm months in pastures of a similar size; our cattle traveled an average of 4.3 km per day with a daily photoperiod exceeding 16 hr in midsummer. Our data do suggest the frequently reported bimodal activity pattern (Figure 3) created by early morning and late afternoon grazing periods (Bailey, Keil, & Rittenhouse, 2004; Lomillos Pérez et al., 2017; Schlecht, Hiernaux, Kadaouré, Hülsebusch, & Mahler, 2006), indicating we are able to detect broad patterns of animal activity from the GPS data alone.

Also consistent in our daily distance data was the increase in prediction error as the interval between logged positions increased. The degradation of accuracy as logging interval increases is frequently reported (Johnson & Ganskopp, 2008; Marcus Rowcliffe et al., 2012; Mills et al., 2006) and, when explicitly quantified, shows an exponential decay that can be corrected by multiplying observed distances by coefficients from the best‐fit line (Akasbi et al., 2012). Our data suffered the same declines, with travel distance underestimations ranging from 15% to 60% depending on logging pattern (burst logging was the worst; Figures 7 and 8). However, when correction factors were determined by maximum likelihood estimation and multiplied by observed distances, predicted daily distances matched 1:1 with actual distances traveled for even novel data analyzed under the burst pattern (Figure 8). Thus, it appears possible to correct distance estimations collected under burst logging if the coefficient can be determined, but we acknowledge this is likely not sufficient for research questions that require accurate estimations of traveled distance.

Although it is frequently reported, traveled distance alone is not necessarily the best measure of animal activity (Ungar et al., 2005). Often, finer‐scale data including instantaneous speed and head position predict individual behavior, and such data are easily obtained from high‐frequency GPS logging intervals and additional sensors such as 3‐D accelerometers (Moreau, Siebert, Buerkert, & Schlecht, 2009). Many commercial systems can be ordered with accelerometers, and Adafruit offers several options that can be integrated with the system we describe here; in fact, we quickly soldered the Adafruit ADXL335 (Table 1) onto a datalogger and updated the program prior to the final round of field trials. However, these raw data are not immediately useful as it is standard procedure among studies reporting accurate predictions of activity and behavior from GPS/accelerometer data to calibrate activity classifications with human observations (e.g., Augustine & Derner, 2013; Schlecht, Hülsebusch, Mahler, & Becker, 2004), but the models are not necessarily complicated. Augustine and Derner (2013) found that binary grazing/not grazing classification had the highest accuracy in modeling cattle activity on rangeland, and distance traveled per 5‐min interval combined with head position data were the two best predictors of grazing activity. The burst logging pattern is ideal for these types of data, providing high‐frequency observations for the duration of the burst. Assuming activity can be sampled at some hourly interval rather than constantly monitored, burst logging is a potential solution for gaining high‐frequency data over long time periods through extended battery life, especially if highly‐accurate estimates of daily travel are not required.

## CONFLICT OF INTEREST

The authors declare no conflict of interest. The authors are affiliated with neither Adafruit Industries nor any other company mentioned herein. All materials were purchased or otherwise procured by the authors.

## AUTHOR CONTRIBUTIONS

DAM designed and built the GPS dataloggers, analyzed data, and was primary author on the manuscript. BG designed the field component, coordinated field work, and edited the manuscript. JWS conducted field work, managed data, and edited the manuscript.
