# Supplementary material for: Assessment of a livestock GPS collar based on an open‐source datalogger informs best practices for logging intensity
Source: Ecol Evol. 2018 May 7;8(11):5649–60. doi: 10.1002/ece3.4094 (PMC6010917; doi:10.1002/ece3.4094)
Supplement: Supplementary file 1 [file ECE3-8-5649-s001.pdf]

Appendix 1

Program for the Adafruit Feather M0 Adalogger with GPS Wing. This sript can be saved with the `.ino` extension and opened with the Arduino IDE. Note that the IDE does not include Adafruit boards by default. This URL explains how they are easily added: https://learn.adafruit.com/adafruit-feather-m0-adalogger?view=all#setup.

Also note that the example files provided by Adafruit are heavily commented for users who wish to understand what the various commands and options mean. We've removed the majority of comments for brevity.

```

#include <Adafruit_GPS.h>
#include <SPI.h>
#include <SD.h>

const int chipSelect = 4;

int attempts, good_fixes, bad_fixes ;

// hardware serial port
#define GPSSerial Serial1
Adafruit_GPS GPS(&GPSSerial);

#define GPSECHO false

uint32_t timer = millis();

void setup()
{
 // while (!Serial);  // uncomment to wait until Serial is ready
 Serial.begin(115200);
  attempts = 0;
  good_fixes = attempts;
  bad_fixes = attempts;

  // Initialization sub-routines defined at end of sketch
  SDcheck() ;
  GPSconnect() ;
  delay(100) ;

}

void loop() {
  char c = GPS.read();
  if (!GPS.fix)  Serial.println("no location! no fix! ");
  if (GPSECHO)
    if (c) Serial.print(c);
   if (GPS.newNMEAreceived()) {
   if (!GPS.parse(GPS.lastNMEA()))
      return;
  }
  if (timer > millis()) timer = millis();
  if (millis() - timer > 20000) {
    timer = millis(); // reset the timer
    attempts = attempts + 1;

  File dataFile = SD.open("log.txt", FILE_WRITE);
   if (dataFile) {
    dataFile.print(GPS.day, DEC); dataFile.print('-');
    dataFile.print(GPS.month, DEC); dataFile.print("-20");
    dataFile.print(GPS.year, DEC);
      dataFile.print(", ");
    //time
    dataFile.print(GPS.hour, DEC); dataFile.print(':');
    dataFile.print(GPS.minute, DEC); dataFile.print(':');
```

```
    dataFile.print(GPS.seconds, DEC);
      dataFile.print(", ");
    //location data
 if (GPS.fix) {
  good_fixes = good_fixes + 1;
    //location data
      dataFile.print(GPS.latitude, 4); dataFile.print(GPS.lat);
      dataFile.print(", ");
    dataFile.print(GPS.longitude, 4); dataFile.print(GPS.lon);
      dataFile.print(", ");
    dataFile.print(GPS.altitude);
      dataFile.print(", ");
    dataFile.print(GPS.speed);
      dataFile.print(", ");
    dataFile.print(GPS.angle);
      dataFile.print(", ");
    dataFile.print((int)GPS.fix);
      dataFile.print(", ");
    dataFile.print((int)GPS.fixquality);
      dataFile.print(", ");
    dataFile.println((int)GPS.satellites);
       dataFile.close();
  }
 else {
   bad_fixes = bad_fixes + 1;
      dataFile.print("na ,");
      dataFile.print("na, ");
      dataFile.print("na, ");
       dataFile.print("na, ");
      dataFile.print("na, ");
     dataFile.print((int)GPS.fix);
      dataFile.print(", ");
    dataFile.print((int)GPS.fixquality);
      dataFile.print(", ");
    dataFile.print((int)GPS.satellites);
          dataFile.println(", ");
       dataFile.close();
   }
         } // close file
     } // close if timer
 } // close loop

void SDcheck (void) {
if (!SD.begin(chipSelect)) {
   Serial.println("Card failed, or not present");
// don't do anything more:
 return;
 }
     Serial.println("card initialized.");
     delay(1000);

  File dataFile = SD.open("log.txt", FILE_WRITE);
  if (dataFile) {
  Serial.println("File open. Logging!");

  delay(1000);
  }
  // If the file is not open, pop up an error
  else {
  Serial.println("No file! Not logging!");
   delay(1000);
   return;
  }
}

void GPSconnect (void) {
  GPS.begin(9600);
  GPS.sendCommand(PMTK_SET_NMEA_OUTPUT_RMCGGA);
```

```
  GPS.sendCommand(PMTK_SET_NMEA_UPDATE_1HZ); // 1 Hz update rate
  GPS.sendCommand(PGCMD_ANTENNA);
  delay(1000);
  // Ask for firmware version
  GPSSerial.println(PMTK_Q_RELEASE);
}
```

Appendix 2

R script used for processing any GPS data collected from the Arduino script in Appendix 1.

```
LongLatToUTM <- function(x,y,zone){
                  require(rgdal)
                     xy <- data.frame(ID = 1:length(x), X = x, Y = y)
                   coordinates(xy) <- c("X", "Y")
                   proj4string(xy) <- CRS("+proj=longlat +datum=WGS84")
                   res <- spTransform(xy, CRS(paste("+proj=utm +zone=",
                                                    zone," ellps=WGS84",sep='')))
                   return(as.data.frame(res))}

# Load required packages
  pacman::p_load(plyr, dplyr, lubridate,
                 sp, rgdal)

setwd("C:/Users/devan.mcgranahan/GoogleDrive/R-duino/GPS collars/data/")

samp.locs <- list.dirs(recursive = FALSE)
samp.rnds <- list.dirs(path=samp.locs, full.names=FALSE, recursive = FALSE)

# Create empty data frame
  GPS.d <- data.frame(date=character(),
                   time=character(),
                   long=character(),
                   lat=character(),
                   elevation=numeric(),
                   speed=numeric(),
                   angle=numeric(),
                   fix=integer(),
                   quality=integer(),
                   num_sats=integer(),
                   location=character(),
                   # round=character(),
                   unit=character(),
                   stringsAsFactors=FALSE)

# Import data from nested directories
  for(i in 1:length(samp.locs)){
    loc = stringr::str_replace_all(samp.locs[[i]],"[[:punct:]]","")
    rnds = list.dirs(path=samp.locs, recursive = FALSE)
    rnd.names <- list.dirs(path=samp.locs, full.names=FALSE, recursive = FALSE)
    for(j in 1:length(samp.rnds)){
      rnd = rnd.names[[j]]
      files = list.files(path=rnds[[j]], pattern=".TXT$")
        for(k in 1:length(files)){
          name = files[[k]]
          fp = file.path(loc, rnd, files[k])
          z <- read.csv(fp, header=FALSE)[1:10]
          d <- as.data.frame(z)
          colnames(d) <- c("date","time","lat","lon","elevation",
                           "speed","angle","fix","quality","num_sats")
          d$location <- loc
```

```
        # d$round <- rnd.names[[j]]
        d$unit <- stringr::str_extract(name, "[[:digit:]]+")
        GPS.d <- rbind(GPS.d, d)
        rm(fp, z, d)
      }
    rm(rnd, files)
  }
  rm(loc)
}


# Some housekeeping
GPS.d <- GPS.d[!duplicated(GPS.d), ] # from 1102968 to 938468
positions <- subset(GPS.d, fix==1)  # removes 7602 of 1102968



# Convert data to numeric. Maintains default projection.
positions$lat <- as.character(positions$lat)
positions$lon <- as.character(positions$lon)
positions <- within(positions,
                lat <- sprintf("%.4f", round(
                  ifelse(substr(lat, nchar(lat), nchar(lat))=="S",
                         as.numeric(substr(lat, 1, nchar(lat)-1))*-1,
                         as.numeric(substr(lat, 1, nchar(lat)-1))),4)))
positions <- within(positions,
                lon <-  sprintf("%.4f", round(
                  ifelse(substr(lon, nchar(lon), nchar(lon))=="W",
                         as.numeric(substr(lon, 1, nchar(lon)-1))*-1,
                         as.numeric(substr(lon, 1, nchar(lon)-1))),4)))

# Convert default long/lat format

positions$lat <- as.numeric(positions$lat)
positions$lon <- as.numeric(positions$lon)
positions <- dim(subset(positions, lat > 0 & lon < 0) )
positions <- droplevels(positions)

geo.d <- data.frame(lat=I(as.character(sprintf("%.6f", positions$lat))),
                 lon=I(as.character(sprintf("%.6f", positions$lon))) )

# UTM conversion. Takes hours on full dataset
for(i in 1:nrow(geo.d)) {
    for(j in 1:ncol(geo.d)) {
 lhs <- substr(geo.d[i,j], 1, nchar(geo.d[i,j])-9)
 rhs <- sprintf("%.6f", round(as.numeric(substr(geo.d[i,j],
                                   nchar(geo.d[i,j])-8,
                                   nchar(geo.d[i,j])))/60,8))
 geo.d[i,j] <- paste(lhs, substr(rhs, 2,
                               nchar(rhs)), sep = "")  }}

geo.d$lat <- as.numeric(geo.d$lat)
geo.d$lon <- as.numeric(geo.d$lon)
```

```r
positions$lat <- geo.d$lat
positions$lon <- geo.d$lon

geo.sp <- subset(positions, lon < -100) # Outlier housekeeping

coordinates(geo.sp) <- c("lon","lat")
proj4string(geo.sp) <- CRS("+proj=longlat +datum=WGS84")

geo.sp <- spTransform(geo.sp, CRS("+proj=utm +zone=13 +datum=NAD83 +units=m
                                  +no_defs +ellps=GRS80 +towgs84=0,0,0"))
rc <- geo.sp[hett.past,]  # crop locations to pasture boundaries

# Connect positions with patch data

patch.dat <- sp::over(rc, hrec.patches)
rc@data <- cbind(rc@data, patch.dat)
rc.df <- data.frame(rc)
rm(geo.sp, rc) # These are very large; remove them from workspace
```

Appendix 3

R script used for analysis of GPS data for specific research objectives of this paper.

```r
# Set custom functions
CompleteCases <- function(data, desiredCols) {
                  completeVec <- complete.cases(data[, desiredCols])
                  return(data[completeVec, ])}


  rc.df <- CompleteCases(rc.df, "Patch") # Further housekeeping
  rc.df <- rc.df[, !(names(rc.df) %in% c("elevation", "fix","num_sats",
                                        "quality", "optional"))]

# Create timestamp
  rc.df$timestamp <- paste(rc.df$date, rc.df$time)
  rc.df$timestamp <- strptime(rc.df$timestamp,
                             format='%d-%m-%Y %H:%M:%S', tz = "MST")
  rc.df$timestamp <- as.POSIXct(rc.df$timestamp)
  rc.df$month <- months(rc.df$timestamp, abbreviate=FALSE)

# Clean up for analysis
  rc.df <- CompleteCases(rc.df, c("unit", "month"))
  rc.df$PastureRep <- tolower(rc.df$PastureRep)
  rc.df$month <- tolower(rc.df$month)
  rc.df$Livestock <- tolower(rc.df$Livestock)

# limit data to units that logged at least 1000 positions per deployment
  counts <- plyr::count(rc.df, vars=c("month","PastureRep","Livestock","unit"))
  low.counts <- counts[counts$freq<1000, ]
  low.rows <- with(low.counts, paste(month, PastureRep,
                                     Livestock, unit))

  rc.df <- rc.df[! with(rc.df, paste(month, PastureRep,
                                     Livestock, unit) %in% low.rows), ]

# Get durations
  durations <- ddply(rc.df, .(month, PastureRep,
                                     Livestock, unit),
                    summarise,
                    duration=difftime(time1=max(timestamp),
                                      time2=min(timestamp),
                                      units="hours"))

# Identify first obs per minute
  rc.df$ts.min <- rc.df$timestamp %>% cut(., breaks="min") %>%
                            as.POSIXct() %>%
                              - lubridate::hours(6)

  # save(rc.df, file="./r objects/rc.df.Rdata")

# Narrow to daylight period (constant logging)
  day.df <- rc.df
  day.df$hms <- format(day.df$timestamp, format = "%H:%M:%S")
```

```r
  day.df$hms <- as.POSIXct(day.df$hms, format = "%H:%M:%S", tz="MsT")
  day.df <- day.df  %>%
              dplyr::filter(hms >= paste(Sys.Date(), "04:00:00") &
                             hms <= paste(Sys.Date(), " 22:00:00") )
  day.df$PastureRep <- tolower(day.df$PastureRep)

  # data.frame with just first observation per minute
     min.df <- day.df[c(TRUE, day.df$ts.min[-1] !=
                         day.df$ts.min[-length(day.df$ts.min)]), ]

# Calculate mean distance among units at the beginning of each minute

  min.mean <- plyr::ddply(.data=min.df, .(month, PastureRep,
                                      Livestock, ts.min),
                         .fun=function(x) {
                          m = cbind(x$lon, x$lat)
                          d = dim(m)[1]
                         mean.dist = ifelse( d >= 2,
                                round(mean(vegan::vegdist(x=m,
                                         method="euc")),1),
                                  "NA" )
                         return(mean.dist) })
  min.mean <- plyr::rename(min.mean, c("V1"="mean.dist"))
  min.mean <- min.mean[! min.mean$mean.dist == "NA", ]
  min.mean$mean.dist <- as.numeric(min.mean$mean.dist)
  min.mean$hm <- format(min.mean$ts.min, format = "%H:%M")
  min.mean$hm <- as.POSIXct(min.mean$hm, format = "%H:%M")
  # save(min.mean, file="./r objects/min.mean.Rdata")

   min.mean.sum <- ddply(min.mean, .(month, Livestock),
                         summarise,
                         dist.mean = round(mean(mean.dist), 1),
                         dist.se = round(sqrt(var(mean.dist)/length(mean.dist)),2))

      min.mean.sum$month <- factor(min.mean.sum$month,
                                     levels = c("june", "july",
                                                 "august", "september"))
       # save(min.mean.sum, file="./r objects/min.mean.sum.Rdata")

#
# Comparing logging intervals
#

# Proportional use for full 40-ac patches

  # Cut to 5- and 10-min intervals
    day.df$PastureRep <- tolower(day.df$PastureRep)
    day.df$ts.5min <- cut(as.POSIXct(day.df$timestamp), breaks="5 min")
    day.df$ts.10min <- cut(as.POSIXct(day.df$timestamp), breaks="10 min")

  # Grab first observation from each interval (regular logging)
    FP.e.5 <- day.df[c(TRUE, day.df$ts.5min[-1] !=
                          day.df$ts.5min[-length(day.df$ts.5min)]), ]
```

```
    FP.e.10 <- day.df[c(TRUE, day.df$ts.10min[-1] !=
                              day.df$ts.10min[-length(day.df$ts.10min)]), ]

  # Grab all observations from first 10-min interval per hour (burst logging)
    FP.a.10 <- day.df  %>%
              dplyr::filter(substr(as.character(ts.10min),15,16) == "07")
    FP.a.5 <- day.df  %>%
              dplyr::filter(substr(as.character(ts.5min),15,16) == "12")

    FP.ints <- list(constant=day.df,
                    e.5=FP.e.5,
                    e.10=FP.e.10,
                    a.5=FP.a.5,
                    a.10=FP.a.10)
    # save(intervals, file="./r objects/intervals.Rdata")

# Calculate proportion use per patch

 FP.ints$constant %>% group_by_at(vars(month, PastureRep, Livestock,
                    unit, PatchNum)) %>%
                   summarize (n = n()) %>%
                   dplyr::mutate(const.prop = round(n / sum(n), 3) ) %>%
                     select(-n) %>%
                          as.data.frame() -> FP.prop.const

  FP.ints$e.5 %>% group_by(month, PastureRep, Livestock,
                    unit, PatchNum) %>%
                   summarise (n = n())%>%
                   dplyr::mutate(reg.prop = round(n / sum(n), 3) ) %>%
                     select(-n) %>%
                          as.data.frame() -> FP.prop.reg.5

 FP.ints$e.10 %>% group_by(month, PastureRep, Livestock,
                    unit, PatchNum) %>%
                   summarise (n = n())%>%
                   dplyr::mutate(reg.prop = round(n / sum(n), 3) ) %>%
                     select(-n) %>%
                          as.data.frame() -> FP.prop.reg.10

 FP.ints$a.5 %>% group_by(month, PastureRep, Livestock,
                    unit, PatchNum) %>%
                   summarise (n = n())%>%
                   dplyr::mutate(burst.prop = round(n / sum(n), 3) ) %>%
                     select(-n) %>%
                          as.data.frame() -> FP.prop.burst.5

 FP.ints$a.10 %>% group_by(month, PastureRep, Livestock,
                    unit, PatchNum) %>%
                   summarise (n = n())%>%
                   dplyr::mutate(burst.prop = round(n / sum(n), 3) ) %>%
                     select(-n) %>%
                          as.data.frame() -> FP.prop.burst.10
```

```r
  FP.patch.props.5 <- merge(x=FP.prop.const, y=FP.prop.reg.5)
  FP.patch.props.5 <- merge(x=FP.patch.props.5, y=FP.prop.burst.5)
  FP.patch.props.5$Period <- "5 min"

  FP.patch.props.10 <- merge(x=FP.prop.const, y=FP.prop.reg.10)
  FP.patch.props.10 <- merge(x=FP.patch.props.10, y=FP.prop.burst.10)
  FP.patch.props.10$Period <- "10 min"

  FP.patch.props <- rbind(FP.patch.props.5, FP.patch.props.10)
  FP.patch.props$`Patch size` <- "16 ha patches"
  FP.patch.props <- plyr::rename(FP.patch.props, c("PatchNum"="Patch"))

# Getting proportional use for tiny patches

  GridPatches.shp <- rgdal::readOGR(dsn="C:/Users/devan.mcgranahan/GoogleDrive/QGIS/
                                  HREC/sampling/GridPatches",
                                      layer="GridPatches")

  day.sp <- day.df[, ! names(day.df) %in% c("speed","angle", "location",
                                          "id","Patch", "hectares",
                                          "last.burn", "status2017") ]
  coordinates(day.sp) <- c("lon","lat")
  proj4string(day.sp) <- CRS("+proj=utm +zone=13 +datum=NAD83 +units=m
                           +no_defs +ellps=GRS80 +towgs84=0,0,0")

  day.sp <- day.sp[GridPatches.shp,]  # crop locations to gridded patches
  # Connect positions with GridPatch data

    day.dat <- sp::over(day.sp, GridPatches.shp)
    day.sp@data <- cbind(day.sp@data, day.dat)
    GP.df <- data.frame(day.sp)
    rm(day.sp, day.dat)

  GP.df <- GP.df %>% dplyr::rename(., GridPatch = id_2)
  GP.df <- GP.df[, ! names(GP.df) %in% c("Id","Current_Tr","Hectares",
                                       "New_Treatm","PastureRep.1","location",
                                       "xmin","xmax", "ymin", "ymax","optional") ]

    GP.e.5 <- GP.df[c(TRUE, GP.df$ts.5min[-1] !=
                          GP.df$ts.5min[-length(GP.df$ts.5min)]), ]
    GP.e.10 <- GP.df[c(TRUE, GP.df$ts.10min[-1] !=
                          GP.df$ts.10min[-length(GP.df$ts.10min)]), ]

  # Grab all observations from first 10-min interval per hour (burst logging)
    GP.a.10 <- GP.df  %>%
                dplyr::filter(substr(as.character(ts.10min),15,16) == "07")
    GP.a.5 <- GP.df  %>%
                dplyr::filter(substr(as.character(ts.5min),15,16) == "12")

    GP.ints <- list(constant=GP.df,
                    e.5=GP.e.5,
                    e.10=GP.e.10,
                    a.5=GP.a.5,
```

```r
                      a.10=GP.a.10)

  GP.ints$constant %>% group_by_at(vars(month, PastureRep, Livestock,
                     unit, GridPatch)) %>%
                    summarize (n = n()) %>%
                    dplyr::mutate(const.prop = round(n / sum(n), 3) ) %>%
                      select(-n) %>%
                           as.data.frame() -> GP.prop.const

  GP.ints$e.5 %>% group_by(month, PastureRep, Livestock,
                     unit, GridPatch) %>%
                    summarise (n = n())%>%
                    dplyr::mutate(reg.prop = round(n / sum(n), 3) ) %>%
                      select(-n) %>%
                           as.data.frame() -> GP.prop.reg.5

 GP.ints$e.10 %>% group_by(month, PastureRep, Livestock,
                     unit, GridPatch) %>%
                    summarise (n = n())%>%
                    dplyr::mutate(reg.prop = round(n / sum(n), 3) ) %>%
                      select(-n) %>%
                           as.data.frame() -> GP.prop.reg.10

 GP.ints$a.5 %>% group_by(month, PastureRep, Livestock,
                     unit, GridPatch) %>%
                    summarise (n = n())%>%
                    dplyr::mutate(burst.prop = round(n / sum(n), 3) ) %>%
                      select(-n) %>%
                           as.data.frame() -> GP.prop.burst.5

 GP.ints$a.10 %>% group_by(month, PastureRep, Livestock,
                     unit, GridPatch) %>%
                    summarise (n = n())%>%
                    dplyr::mutate(burst.prop = round(n / sum(n), 3) ) %>%
                      select(-n) %>%
                           as.data.frame() -> GP.prop.burst.10

  GP.patch.props.5 <- merge(x=GP.prop.const, y=GP.prop.reg.5)
  GP.patch.props.5 <- merge(x=GP.patch.props.5, y=GP.prop.burst.5)
  GP.patch.props.5$Period <- "5 min"

  GP.patch.props.10 <- merge(x=GP.prop.const, y=GP.prop.reg.10)
  GP.patch.props.10 <- merge(x=GP.patch.props.10, y=GP.prop.burst.10)
  GP.patch.props.10$Period <- "10 min"

  GP.patch.props <- rbind(GP.patch.props.5, GP.patch.props.10)
  GP.patch.props$`Patch size` <- "1 ha patches"
  GP.patch.props <- subset(GP.patch.props, const.prop!=0)
  GP.patch.props <- plyr::rename(GP.patch.props, c("GridPatch"="Patch"))

# Combine the two patch type datasets

  patch.props <- rbind(FP.patch.props, GP.patch.props)
```

```
# Proceed with analysis

  patch.props$`Regular` <- with(patch.props, reg.prop - const.prop)
  patch.props$`Burst` <- with(patch.props, burst.prop - const.prop)

  patch.props.st <- data.frame(patch.props[,c(1:5,9:10)], stack(patch.props[11:12]))
  colnames(patch.props.st)[8:9] <- c("Difference", "Logging pattern")

  patch.props.sum <- ddply(patch.props.st, .(month,  Livestock, PastureRep, Period,
                                            `Logging pattern`, Patch.size, unit),
                           summarise,
                           diff.max = round(max(abs(Difference)), 3))
    patch.props.sum <- ddply(patch.props.sum, .(month,  Livestock, Period,
                                            `Logging pattern`, Patch.size),
                           summarise,
                           diff.mean = round(mean(abs(diff.max)), 3),
                           diff.se = round(sqrt(var(abs(diff.max))/
                                                 length(abs(diff.max))),3)     )

  patch.props.sum$month <- factor(patch.props.sum$month,
                                  levels = c("june", "july",
                                             "august", "september"))
   # save(patch.props.sum, file="./r objects/patch.props.sum.Rdata")

#
# Comparing total distance travelled
#

const.dat <- with(intervals$constant, data.frame(lon, lat, month, date,
                                                PastureRep, Livestock, unit,
                                                type="Constant", Period="20 sec"))
e5 <- with(intervals$every.5, data.frame(lon, lat, month, date,
                                                PastureRep, Livestock, unit,
                                                type="Regular", Period="5 min"))
e10 <- with(intervals$every.10, data.frame(lon, lat, month, date,
                                                PastureRep, Livestock, unit,
                                                 type="Constant", Period="10 min"))
a5 <- with(intervals$all.5, data.frame(lon, lat, month, date,
                                                PastureRep, Livestock, unit,
                                                type="Burst", Period="5 min"))
a10 <- with(intervals$all.10, data.frame(lon, lat, month, date,
                                                PastureRep, Livestock, unit,
                                                 type="Burst", Period="10 min"))

dist.dat <- rbind(const.dat, e5, e10, a5, a10)


daily.dists <- plyr::ddply(subset(dist.dat, month!="june"),
                                    .(month, date, PastureRep,
                                      Livestock, unit, type, Period),
                .fun=function(x) {
          coordinates(x) <- c("lon","lat")
          proj4string(x) <- CRS("+proj=utm +zone=13 +datum=NAD83 +units=m
```

```
                                 +no_defs +ellps=GRS80 +towgs84=0,0,0")
          x <- spTransform(x, CRS("+proj=longlat +datum=WGS84"))

          round(sum(sapply(seq_along(x[-1, ]), function(i)
                  geosphere::distGeo(p1 = x[i, ], p2 = x[i+1, ],
                                    a=6378137, f=1/298.257223563))),1)
              } )

daily.dists <- daily.dists %>% dplyr::rename(., total.dist = V1)

dist.diffs <- with(distances, data.frame(month, date, PastureRep,
                                          Livestock, unit,
                                          a10.diff = ((const.dist-a10.dist)/const.dist),
                                          e10.diff = ((const.dist-e10.dist)/const.dist),
                                          a5.diff = ((const.dist-a5.dist)/const.dist),
                                          e5.diff =((const.dist-e5.dist)/const.dist)) )


dist.diffs.st <- data.frame(dist.diffs[,c(1:5)], stack(dist.diffs[6:9]))
colnames(dist.diffs.st)[6:7] <- c("underestimation", "id")

dist.diffs.st$Period <- dist.diffs.st$id %>%
                            plyr::revalue(.,
                                         c("a10.diff"="10 min",
                                           "a5.diff"="5 min",
                                           "e10.diff"="10 min",
                                           'e5.diff'="5 min"))

dist.diffs.st$`Logging pattern` <- dist.diffs.st$id %>%
                            plyr::revalue(.,
                                         c("a10.diff"="Burst",
                                           "a5.diff"="Burst",
                                           "e10.diff"="Regular",
                                           'e5.diff'="Regular"))

dist.diffs.sum <- ddply(dist.diffs.st, .(month,  Livestock,
                                         Period, `Logging pattern`),
                           summarise,
                           diff.mean = round(mean(underestimation), 3),
                           diff.se = round(sqrt(var(underestimation)/
                                                  length(underestimation)),3)    )

#
# Linear modeling
#

# Testing distance differences among categorical variables

  glmm0 <- lme4::glmer(underestimation ~ 1 + (1|PastureRep/unit/month),
                      family="binomial", dist.diffs.st)
  glmm1 <- lme4::glmer(underestimation ~ Livestock +
                         (1|PastureRep/unit/month),
                      family="binomial", dist.diffs.st)
```

```
  anova(glmm0, glmm1) # No difference

   glmm2 <- lme4::glmer(underestimation ~ `Logging pattern` +
                            (1|PastureRep/unit/month),
                        family="binomial", dist.diffs.st)
  anova(glmm0, glmm2) # Different

  glmm3 <- lme4::glmer(underestimation ~ `Logging pattern` + Period +
                           (1|PastureRep/unit/month),
                         family="binomial", dist.diffs.st)
  anova(glmm2, glmm3) # Different

# Maximum Likelihood Estimation

  mle.mod.terms <- data.frame(pattern=character(),
                              model=character(),
                              slope.est=numeric(),
                              slope.ciL=numeric(),
                              slope.ciU=numeric() )

  mod.names <- names(distances[7:10])

  for(i in 1:length(mod.names)) {

    X = noquote(c(mod.names[i]) )

    # set data paramaters
    y = distances$const.dist
    x = eval(parse(text=noquote(paste("distances$",X,sep="") )  ) )
    mu = round(mean(x),0)
    sigma = round(sd(x),1)
    # Set regression parameters
      # define and fit linear model
        orig.form = paste("y ~ ",X,sep = " ")
        orig.form <- as.formula(orig.form)
        org.mod <- lm(formula=orig.form, data=distances)
      # use regression coefficients as starting points for mle
        B0 = round(as.numeric(coef(org.mod) [1] ),1)
        B1 = round(as.numeric(coef(org.mod) [2] ),2)
    # define log-likelihood function
    LL <- function(beta0, beta1, Mu, Sigma) {
              R =  y - x  * beta1 - beta0
              R = suppressWarnings(dnorm(R, Mu, Sigma, log = TRUE))
              -sum(R)
        }
    require(bbmle)
    fit <- mle2(LL, start = list( beta0 = B0, beta1 = B1,
                                  Mu = mu, Sigma = sigma),
                method="L-BFGS-B")
    mle.CIs <- suppressWarnings(confint(fit))

    do <- data.frame(pattern=X,
                model="Uncorrected",
```

```r
              slope.est = as.numeric(fit@coef[2] ),
              slope.ciL = as.numeric(mle.CIs[2,1]),
              slope.ciU = as.numeric(mle.CIs[2,2]) )
  # fit June data
  cor.mod <- lm(y ~ I(x*do$slope.est), data=june.dists )
  cor.CIs <- confint(cor.mod)
  dc <- data.frame(pattern=X,
                model="Corrected",
                slope.est = as.numeric(coef(cor.mod) [2] ),
                slope.ciL = as.numeric(cor.CIs[2,1]),
                slope.ciU = as.numeric(cor.CIs[2,2]) )
  d <- rbind(do, dc)
  mle.mod.terms <- rbind(mle.mod.terms, d)
  }
```
